# Understanding Key Factors Influencing Consumers’ Willingness to Try, Buy, and Pay a Price Premium for Mycoproteins

**DOI:** 10.3390/nu14163292

**Published:** 2022-08-11

**Authors:** David Dean, Meike Rombach, Wim de Koning, Frank Vriesekoop, Wisnu Satyajaya, Puspita Yuliandari, Martin Anderson, Philippe Mongondry, Beatriz Urbano, Cristino Alberto Gómez Luciano, Wendy Hao, Emma Eastwick, Elma Achirimbi, Zheng Jiang, Anouk Boereboom, Farzana Rashid, Imran Khan, Beatriz Alvarez, Luis Kluwe Aguiar

**Affiliations:** 1 Faculty of Agribusiness and Commerce, Lincoln University, Lincoln P.O. Box 85084, Canterbury, New Zealand; 2 Food Land and Agribusiness Management Department, Harper Adams University, Newport, Shropshire TF10 8NB, UK; 3 Department of Food Technology, HAS University of Applied Science, P.O. Box 90108, 5200 MA Hertogenbosch, The Netherlands; 4 Department of Agricultural Technology, Lampung University, Bandar Lampung 35145, Indonesia; 5 Department of Food, Technology & Bioresource Science, Groupe ESA, 49007 Angers, France; 6 Department of Agricultural and Forrest Engineering, University of Valladolid, 47002 Valladolid, Spain; 7 Specialized Institute of Higher Studies Loyola, San Cristóbal 91000, Dominican Republic; 8 Department of Zoology, Lahore College for Women University, Lahore 54600, Pakistan; 9 Department of Human Nutrition, The University of Agriculture, Peshawar 25120, Pakistan; 10 Faculty of Chemistry, Autonomous University of Queretaro, Queretaro 76017, Mexico

**Keywords:** mycoprotein, PLS-SEM, preferences, meat alternatives, fungal proteins

## Abstract

Mycoprotein is a fungal-based meat alternative sold in food retail in various countries around the world. The present study builds on a multi-national sample and uses partial least square structural equation modeling. The proposed conceptual model identified key factors that are driving and inhibiting consumer willingness to try, buy, and pay a price premium for mycoprotein. The results relate to the overall sample of 4088 respondents and to two subsample comparisons based on gender and meat consumption behavior. The results show that the biggest drivers of willingness to consume mycoprotein were healthiness, followed by nutritional benefits, safe to eat, and sustainability. Affordability and taste had mixed results. Willingness to consume mycoprotein was inhibited if nutritional importance was placed on meat and, to a lesser extent, if the taste, texture, and smell of meat were deemed important. Best practice recommendations address issues facing marketing managers in the food industry.

## 1. Introduction

In the last decade, consumer demand for sustainably and ethically produced food has led to a shift in lifestyle and dietary patterns in several Western societies [1,2,3]. Meat consumption all around the world has been steadily increasing, reaching 324 million metric tons in 2020 [4]. The increase in meat demand has led to negative societal and environmental externalities such as deforestation and greenhouse emissions [5,6,7]. These externalities are often cited as consumer motivations towards meat-reduced lifestyle changes as well as concerns for animal welfare and cruelty, and health-related reasons [8,9].

Diets devoid of meat, such as vegetarianism and veganism, are increasing in popularity [10,11,12]. A vegetarian diet requires the non-consumption of meat, seafood, or any form of animal flesh, while a vegan diet requires abstaining from eating not only meat but also any animal-based product, for instance, milk, yogurt, eggs, or honey [13,14,15]. The occurrence of meat-reduced or meat-free dietary changes is reflected in the increased availability of plant-based meat, dairy, and egg alternatives in food retail, as well as in the occurrence of vegetarian and vegan options in restaurants [16,17,18]. Currently, soy, wheat, and peas dominate the plant-based meat market, and cultured meat constitutes an emerging product that has enjoyed significant notoriety [8,19].

Cultured meat and many plant-based meat alternatives have been explored in the existing body of literature [8,20,21,22], along with the attitudes, norms, values, motivations to become vegetarian or vegan, health behavior, and dietary beliefs [16]. One meat alternative that has not yet received wide academic attention in the consumer context is mycoprotein [23,24], even though mycoprotein is commercially available and enjoying popularity in consumer markets [24]. Mycoprotein is a fungal-based protein source that was commercially developed in the 1980s and is derived from *Fusarium venenatum*, a fungus belonging to the mold family [25,26]. Quorn is a famous mycoprotein product available in many consumer markets around the world [26,27], which is produced through fermenting fungi spores along with glucose and other nutrients [25]. Until 2020, Quorn had a rather exclusive position and, in many consumer markets, was the only fungal-based protein option available [28]. However, the Swedish business Mycorena developed an alternative product for the European markets with the brand Promyc. Promyc is advertised to be neutral in taste and therefore suitable for a wide variety of products such as burgers, nuggets, protein bars, and snacks [28]. Consumers appreciate mycoprotein products such as Quorn and Promyc for being high in fiber, low in fat, sodium, and sugar, and rich in essential amino acids, and for their meat-like texture. In addition, compared to regular meat production, mycoprotein has a smaller water footprint and smaller carbon emissions [29].

Mycoprotein studies have largely focused on production and processing [25,26,27,29] and, in the consumer area, on consumers’ attitudes, knowledge, and willingness to try [23,24]. However, the willingness to buy and pay a price premium for mycoprotein specifically is largely unexplored. In addition, differences between consumers with varying levels of meat consumption have yet to be investigated. Therefore, the present study is dedicated to this literature gap and explores key factors influencing consumers’ willingness to try, buy, and pay a price premium for mycoprotein for consumers with varying types of meat consumption. In the remainder of this section, these key factors are presented as well as the resultant hypotheses, and these are drawn together to form a conceptual model.

## 2. Health and Safety Aspects of Mycoproteins

Mycoproteins are known to be a high-quality and high-protein source of fungal biomass as they are rich in fibers and low in terms of fat content [30,31]. While dietary studies show that mycoproteins can positively affect appetite regulation, medical studies provide evidence that consumers of mycoproteins have a lower risk of cardiovascular diseases and strokes, and that this form of protein is beneficial in controlling glycemic responses [30]. In addition to these benefits, recent studies have also reported negative health effects. A study related to the Quorn brand emphasized that mycoprotein can cause allergic reactions such as urticaria and anaphylaxis, and severe gastrointestinal issues [32]. For these reasons, mycoproteins are not unilaterally safe for health. Before producers can introduce new mycoprotein products into the market, toxicity testing is required [30]. Amidst this background, the following hypotheses are proposed:

**Hypothesis** **(H1).** *The healthiness of mycoprotein positively impacts willingness to (a) try, (b) buy, and (c) pay a price premium for mycoprotein.*

**Hypothesis** **(H2).** *Food safety positively impacts willingness to (a) try, (b) buy, and (c) pay a price premium for mycoprotein.*

**Hypothesis** **(H3).** *The nutritional benefits of mycoprotein positively impact willingness to (a) try, (b) buy, and (c) pay a price premium for mycoprotein.*

Consumers expect meat alternatives such as mycoproteins to be similar to traditional meat products in terms of appearance and taste. A study on meat alternatives compared consumer preferences for various vegetarian and vegan brands [33]. The results emphasized that Quorn was preferred over other brands, in particular for the product attributes appearance and taste. For the overall consumer preference ranking, Quorn outranked Tivall vegan and vegetarian products, vegan products from Vivera, and Goodbit chicken-style products [33]. However, studies that included in-home usage tests showed that, after several weeks of consumption, there were no significant preference differences between the products. For this reason, the following hypothesis is proposed [33,34,35]:

**Hypothesis** **(H4).** *Taste impacts willingness to (a) try, (b) buy, and (c) pay a price premium for mycoprotein.*

Mycoprotein products such as Quorn are sold as meat substitutes and are often more expensive than traditional meat products [36,37]. These products target consumers who follow a vegetarian, flexitarian, vegan, or other health-conscious lifestyle and are likely to be willing and able to pay a price premium for these alternative proteins [38]. Some studies indicate that consumers are willing to pay a premium for mycoproteins [38], while others emphasize price/demand elasticities [39].

**Hypothesis** **(H5).** *The affordability positively impacts willingness to (a) try, (b) buy, and (c) pay a price premium for mycoprotein.*

Mycoproteins are currently appreciated by consumers favoring sustainability [31,40]. Compared to regular meat products, mycoproteins are more sustainable due to the negative externalities associated with meat production, namely, concerns about animal husbandry, inefficient use of resources, and high carbon and water footprints [31]. In this context, consumers focusing on sustainable diets are likely to appreciate Quorn as a third-party verified and certified producer, having obtained the carbon trust footprint [41].

**Hypothesis** **(H6).** *The product’s sustainability positively impacts willingness to (a) try, (b) buy, and (c) pay a price premium for mycoprotein.*

Not only are the product attributes of mycoprotein as a meat alternative crucial to consumer willingness to try, buy, and pay a price premium, but its positioning as a suitable substitute for meat in terms of sensory attributes and nutrition is also important. When buying or eating meat, consumers evaluate meat attributes that are inherent to the product and essential to the sensory experience such as freshness, taste, tenderness, and texture [21]. Accordingly, these attributes are the standard of comparison either in a negative or positive manner, depending on whether consumers appreciate or reject meat. For example, those who feel that only meat provides some nutrients that are necessary for a healthy diet will be less likely to look for substitutes. Likewise, consumers who appreciate the sensory aspects of meat will be likely to favor substitutes that have meat-like aspects, while these aspects may not be appreciated by those who do not appreciate the taste, texture, or smell of meat [34,35]. Many meat substitutes are processed into burger patties or nuggets, and many meat alternatives have tried to mimic the sensory features of meat [34].

**Hypothesis** **(H7).** *The sensory importance of meat negatively impacts willingness to (a) try, (b) buy, and (c) pay a price premium for mycoprotein.*

**Hypothesis** **(H8).** *The nutritional importance of meat negatively impacts willingness to (a) try, (b) buy, and (c) pay a price premium for mycoprotein.*

**Figure 1:**
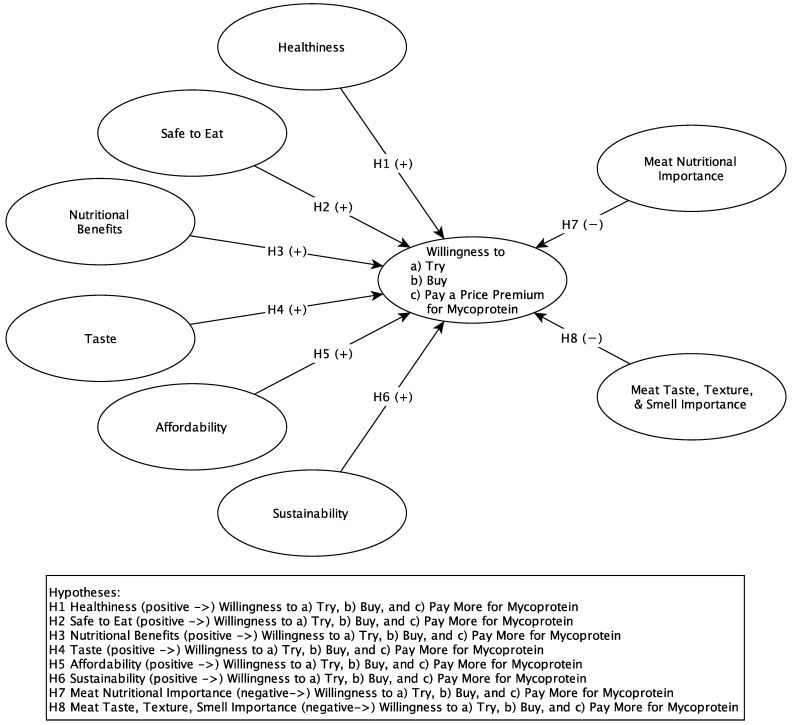
Conceptual Model.

The proposed conceptual model is based on the recent body of literature and is depicted in Figure 1. The conceptual model indicates that willingness to try, buy, and pay a price premium for mycoprotein is driven by the consumers’ perception of product attributes such as healthiness, food safety, nutritional properties, taste, price, and sustainability. Conversely, willingness to try, buy, and pay a price premium is inhibited by the importance placed on the sensory and nutritional of aspects of meat, as mycoprotein is positioned as a meat substitute. In addition, the recent body of literature emphasizes that the perception of factors driving the consumption of meat alternatives as well as attitudes towards meat alternatives is rather diverse. Differences among consumers with varying degrees of meat-eating behavior, as well as those related to gender, have been found. Given that findings related to mycoproteins in this context are relatively scarce, the present study draws from research on plant-based meat alternatives. It is suggested that the appeal of meat alternatives is greater for women than for men [1,42], and very recent studies have found that women consume plant-based meat alternatives more frequently than men [43]. Even associations with meat have been found to differ between men and women [1]. Women tend to associate animals with living beings, note their suffering, and moderate their meat consumption accordingly, while men associate animals with food products, focusing on form and taste. Overall, men tend to have more positive associations with meat compared to women [1]. Plant alternatives are more widely purchased and eaten by consumers with flexitarian, vegetarian, and vegan diets compared to consumers with an omnivore diet [1,11,44]. Therefore, the proposed model will be applied to subgroups and examine whether the hypothesized relationships apply to males, females, vegetarians, flexitarians, and omnivores.

## 3. Materials and Methods

### 3.1. Data Collection and PLS-SEM Approach

An online survey dedicated to meat substitutes such as mycoproteins was administered in 2018/2019 in twelve countries. In China, the USA, France, the United Kingdom, New Zealand, the Netherlands, Brazil, Spain, the Dominican Republic, Mexico, Indonesia, and Pakistan, email and social media links were used as the means to distribute the survey. All survey participants had to be 18 years or older to complete the survey. In addition, survey participants were required to self-report their meat consumption habits. The survey was initially designed in English and subsequently translated into other languages. The survey translation was executed by the co-authors of this paper, as these researchers are native speakers of their various mother tongues and use English as their professional academic language. The approach to translation guaranteed culturally appropriate use of language and translation accuracy. For the English-speaking countries, colloquial adjustments were made. The current research used part of a larger omnibus survey consisting of 99 questions overall (including demographic indicators). Eighteen of the questions were used in the present paper as they were specifically dedicated to the mycoprotein context. The other (unused) questions focused on other meat substitutes including insects, cultured muscle, and plant-based meat alternatives such as tofu. The questions used in the present analysis are provided in the Supplementary Materials. Items and scales were adopted and adapted from the recent body of literature on plant-based and fungal-based meat alternatives. All scale items either used a five-point Likert scale ranging from “strongly disagree (1)” to “strongly agree (5)”, or a “no (1)”, “possible (2)”, or “yes (3)” for willingness to try, buy, and pay a price premium for the product.

A total of 4488 responses were available after data cleaning for the analysis. The software packages SPSS and SmartPLS were used to facilitate the analysis. SPSS was used for the sample descriptive statistics characterizing the background of the survey participants. SmartPLS was used to examine the research model and test the proposed hypotheses using partial least squares structural equation modeling (PLS-SEM) [45]. PLS-SEM is a variance-based approach to structural equation modeling, which is suitable for exploratory studies such as the present one, where the study aims to identify key driver constructs [45,46]. The PLS-SEM approach is particularly suitable as it does not require data to be normally distributed and can accommodate models with multi-item and single-item measures. For complex empirical models, PLS-SEM is appropriate as it ensures a robust prediction in the context of an asymmetric distribution and interdependent observations [45,47]. PLS-SEM is based on three forms of analysis, namely, path analysis, principal component analysis, and regression analysis. The analysis and interpretation of a PLS-SEM model follow a two-stage approach: the assessment of the measurement models and the structural model [46]. The measurement model is dedicated to relationships between the observed data and the latent variables, whereas the structural model focuses on any existing relationships between the latent variables [45,46].

### 3.2. Data Analysis

Table 1 displays the demographic backgrounds of the survey participants. The sample consisted of 63.6% men and 35.8% women, with the remaining 0.6% preferring not to reveal their gender identity. The mean age of the sample was 33.2 years old. In terms of meat-eating behavior, 9.1% of the survey participants indicated eating no meat as they follow a vegetarian lifestyle, and 21.1% reported following a flexitarian lifestyle and eating meat in moderation, whereas most participants at 69.8% indicated being an omnivore. Overall, the US, the Netherlands, and the UK have the highest self-reported percentage of no meat-eating behavior. Table 2 reports the means, minima, maxima, and standard deviations and subgroup means for the single-item measures in the model.

### 3.3. Measurement Model

Hair et al. (2022) indicated that reliability and validity checks are required to assess the measurement model [45]. This is executed by examining factor loadings, Cronbach’s alpha, composite reliability (CR), and the average variance extracted (AVE) of the multi-item scales. For the analysis via the measurement model, construct reliability is considered, which is deemed satisfactory when Cronbach’s alpha and composite reliability are greater than 0.6 [45,46]. Convergent validity is reached when items contribute to constructs and these constructs capture item variation. The contribution of items is examined via factor loadings on the appropriate construct [46]. Following Hair (2022), loadings must be greater than 0.4 [45]. Likewise, a construct is said to capture sufficient item variation when the average variance extracted (AVE) is greater than 0.6 [45]. Table 3 shows that all Cronbach alpha and composite reliability indicators were above the required 0.6 minimum threshold, confirming construct reliability. The average variance extracted (AVE) was higher than 0.5, and factor loadings of all items were higher than 0.6. As shown in Table 3, all the composite reliability values indicate good internal consistency reliability, and all latent variables fulfilled the threshold value and were therefore considered to fulfill the standard recommended for convergent validity.

To assess discriminant validity, checking the Fornell–Larcker criterion and the heterotrait–monotrait ratio of correlations criterion (HTMT) is required [45,47,48]. To satisfy the Fornell–Larcker criterion, each construct’s AVE needs to have a square root that is higher than its correlation with another construct [45,48]. The HTMT examines the correlations of items within a scale and the correlations between items of different scales, which then enables a ratio to be calculated. If this HTMT ratio is less than 0.9, discriminant validity can be confirmed [45]. The variance inflation factor (VIF) determines whether multicollinearity within the data is an issue and is used when target thresholds are less than 5 [45,46]. As shown in Table 4, the discriminant validity requirements were fulfilled for all constructs. All HTMT ratios were below 0.90, and for the Fornell–Larcker criterion, the cross-loadings were less than the diagonal values [45,48,49]. The averaged variance inflation factor (VIF) score was used to determine if multicollinearity affected the model [46]. The VIF scores ranged from 1.408 to 3.606, with an average VIF score of 2.229, indicating that collinearity was not an issue within the proposed model.

### 3.4. Structural Model

In the next step of the analysis, the structural model was assessed, and the proposed hypotheses were examined. This was executed via bootstrapping (5000 iterations), a non-parametric procedure allowing significance testing of estimated path coefficients and relationships between variables [45]. The goodness of fit, explanatory power, and predictive relevance are necessary to evaluate the structural model [45,46]. The proposed structural model was tested, resulting in a goodness of fit (GoF) of 0.522, a normal fit index (NFI) of 0.938, and a standardized root mean square residual (SRMR) of 0.021 for the overall sample (see Table 5). This indicates an adequate model fit, considering that a satisfactory SRMR is lower than 0.08. Values greater than 0.10 are considered problematic, as suggested by Hair et al. (2022) [45]. The model fit scores for all the analyzed subsamples were also adequate, as shown in Table 5.

For the explanatory power, the model’s constructs contributed to an R^2^ of 0.316 for willingness to try mycoprotein, 0.264 for willingness to buy mycoprotein, and 0.237 for willingness to pay a price premium, explaining 31.6% of the variance of willingness to try mycoprotein, 26.4% of the variance of willingness to buy mycoproteins, and 23.7% of the variance of willingness to pay a price premium for mycoproteins. The R^2^ values indicate that the model appears to be slightly better suited to explaining behavior requiring a lower commitment from the consumers such as willingness to try, compared to moderate- or high-commitment behavior such as willingness to buy or to pay a price premium. The latter findings are unsurprising given the relatively high price point of mycoproteins. Even though the R^2^ values in the present model would be classified as weak, given the exploratory nature of the research, the results do provide sufficient explanatory power. It is also important to note that the explanatory power was adequate for all the subgroups, and strongest for the vegetarians.

Predictive relevance was tested using the Stone–Geisser criterion Q^2^. Q^2^ values higher than zero indicate good predictive validity, values higher than 0.25 indicate medium predictive relevance, and values higher than 0.50 indicate strong predictive relevance [45]. The Q^2^ values for the overall sample and subsamples were all above zero, indicating at least adequate predictive relevance, with medium predictive relevance for the overall model (0.268), male subsample (0.288), and vegetarian subsample (0.366).

## 4. Results and Discussion

**Figure 2:**
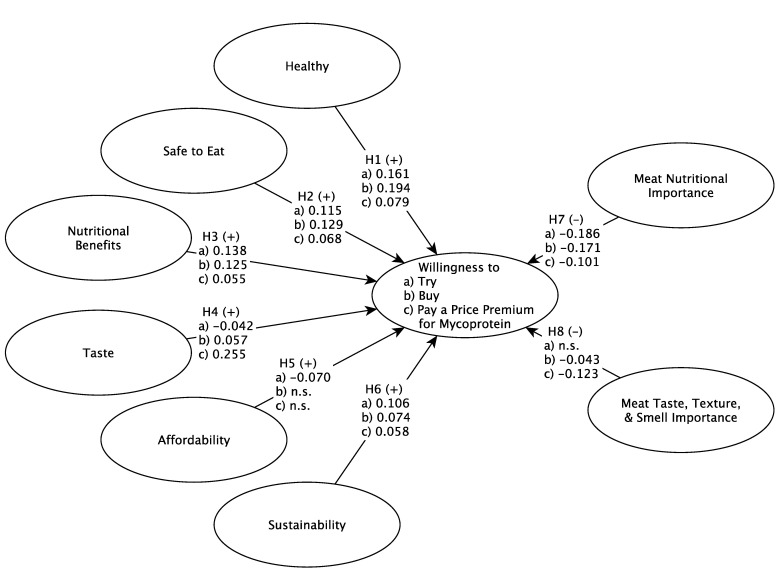
Conceptual Model Results (Overall Sample).

In the overall sample, the healthiness of mycoproteins positively influenced consumers’ willingness to try, buy, and pay a price premium for the product (see Table 6 and Figure 2), supporting hypotheses H1a/b/c. These findings confirm the general consensus in the recent body of literature describing mycoproteins as a product that is bought for its health beneficial characteristics, e.g., dense in fiber and low in fat [30,31,32]. Comparisons between subgroups confirmed a few differences in the subgroups, namely, that H1a/b/c were supported in the male and the omnivore subgroup but not supported in the vegetarian subgroup. While in the recent body of literature, men are described as strong meat eaters [50], the mean age ranging from 23 to 44 years old (see Table 1) indicates that males may be more health-conscious as they belong to cohorts of millennials and Gen Z [43,51]. Both cohorts are open to eating alternatives and interested in healthy lifestyles [43,51]. In the female subgroup, a non-significant relationship was found for willingness to try (H1a); however, the relationships for willingness to buy and pay a price premium were found to be significant (H1b/c). Similarly, in the flexitarian subgroup, the relationship for willingness to buy was not found to be significant (H1b).

However, willingness to try and willingness to pay a price premium were found to have significant relationships, confirming hypotheses H1a and H1c. Given that consumers with a vegetarian and flexitarian lifestyle are eating no meat or only in moderation, they may feel that their plant-based diet is very healthy and do not see meat alternatives such as mycoproteins as a healthier option. After all, mycoproteins are sold in food retail at a high price point targeting vegan, vegetarian, and flexitarian consumers [36,37,38]. In terms of food safety, consumers seem to perceive mycoproteins as safe to eat. In the overall sample, food safety positively influenced consumers’ willingness to try, buy, and pay a price premium, supporting hypotheses H2a/b/c. In all subgroups, a significant relationship for willingness to try and buy was found, confirming hypotheses H2a/b. However, non-significant relationships were found in the female and flexitarian subgroups for willingness to pay a price premium, therefore confirming partial support for hypothesis H2c. Food safety is a basic requirement of any product sold in food retail. However, there are discussions in the recent body of literature on mycoprotein whether the fungal-based meat alternative fulfills this requirement [31,32,33]. While some studies report that the product is safe for consumers, others report consumers having adverse reactions such as nausea, vomiting, and diarrhea after consuming the product. It is even possible to develop allergies over time [31]. The individuals in the subgroups indicating non-significant relationships may be aware of these safety issues and unwilling to pay a price premium.

Overall, the nutritional benefits of mycoprotein are a significant driver of willingness to try, buy, and pay a price premium, supporting H3a/b/c. However, for flexitarians and vegetarians, there was little or no support for nutrition as a driver. Perhaps, like healthiness, flexitarians and vegetarians are already enjoying a plant-based diet that meets their nutrition needs and do not view mycoprotein as a more nutritious option. Certainly, omnivore consumption is driven by the healthiness of mycoproteins (H3a/b/c supported), perhaps because it offers the nutritional benefits of fungal-based protein in forms/textures that are familiar to meat eaters (e.g., burger patties, nuggets, and schnitzels).

In the overall sample, taste positively influenced consumers’ willingness to buy and pay a price premium, but taste was a significant inhibitor of trying mycoproteins, thus supporting hypotheses H4b/c but not H4a. For the subgroups, taste was not a significant driver, and for females, it was a significant inhibitor of willingness to try. For all but the vegetarian and flexitarian subgroups, taste drove willingness to buy and pay a price premium, and in all but the vegetarians, taste drove willingness to pay a price premium. In other words, no one seems to be drawn to trying mycoproteins because of their taste, but presumably after trying, taste is a significant driver for buying and the strongest driver for paying a premium for them. Especially for omnivores and men, or men that love meat [43], the comparability in terms of taste may not be a major concern for trying mycoproteins, but it may be for behavior involving higher commitment such as repeated purchases or paying a price premium. Vegetarians—at least the ones following these diets for concerns related to animal cruelty—may stand at the other end of the continuum and prefer a taste and a sensory experience that diverge from those of meat [1,21].

The affordability, or lack thereof, seems to inhibit most from trying mycoproteins and has no significant impact on willingness to buy them. In the overall sample and in all subgroups apart from flexitarians, only one willingness to try relationship was found to be significant, but it was opposite to what was proposed in hypothesis H5a. In the recent body of literature, the fact that mycoproteins are more costly than meat or dairy and not necessarily affordable for consumers with a low income is a major criticism of this meat alternative [36,39].

Consuming sustainable product options is important for many consumers. In the overall sample, sustainability positively influenced consumers’ willingness to try, buy, and pay a price premium for mycoprotein, confirming hypotheses H6a/b/c. In fact, sustainability was a significant driver for all the subgroups to try mycoproteins, and for all but the female subgroup in willingness to buy mycoproteins. However, for willingness to pay a premium, it was a driver for only the males and flexitarians. Perhaps the sustainability credentials encourage consumers to try and buy mycoproteins, but they are not strong enough to justify a premium. For example, mycoprotein sold under the brand name Quorn is a sustainable product and certified as such [41], but consumers may not be aware that it is, or may not perceive it to be, more sustainable than other products. The certification for Quorn relates to its carbon footprint [41]. Some consumers may be in favor of other aspects of sustainability or want only products that consider all three pillars of sustainability.

Some consumers cannot imagine a diet without meat. Some think that meat is nutritionally necessary or important to maintain a healthy diet. Others are drawn to the sensory aspects of eating meat. For these consumers, finding a substitute for meat is not going to be a high priority, and this was confirmed in the results. In the overall sample, meat’s nutritional importance inhibited consumer willingness to try, buy, and pay a price premium for mycoprotein, confirming hypotheses H8a/b/c. This was also found to be the case for almost all subgroups. This finding is unsurprising given that meat is such an essential part of an omnivore’s diet [21]. The importance of the sensory aspects of meat (taste, texture, smell) was less consistent. Overall, the importance of meat sensory aspects did not significantly influence willingness to try mycoprotein, but it inhibited willingness to buy and pay a premium for mycoproteins, supporting H8b/c but not H8a. For willingness to buy, it was only an inhibitor for males and omnivores, and it inhibited willingness to pay a price premium for males, females, and omnivores.

## 5. Conclusions

Overall, the attributes that were the biggest drivers of willingness to consume mycoprotein were healthiness, followed by nutritional benefits, safe to eat, and sustainability. Affordability and taste had mixed results. Willingness to consume mycoprotein was inhibited if nutritional importance was placed on meat and, to a lesser extent, if sensory aspects of meat (taste, texture, and smell) were deemed important. Of the 21 relationships that were significant in the total sample, 20 were significant for men, 18 for omnivores, 15 for women, 11 for flexitarians, and only 9 for vegetarians. Additionally, the biggest driver was healthiness for men and omnivores, nutritional benefits for women, and safe to eat for flexitarians and vegetarians.

These findings are of relevance to several participants in the food industry, particularly marketing managers in food retail and businesses involved in the production of meat alternatives, as well as non-profit organizations advertising for health and sustainability. Non-profit organizations could be investing in awareness campaigns and best practice advice related to sustainable, balanced, or meat-free diets, promoting mycoproteins and other meat alternatives. Campaigns should strongly focus on sustainability, nutrition, and healthiness, as these are important key factors driving consumers’ willingness to try, buy, and pay a price premium. To stand out and to be genuine, organizations should point out the relatively high price point and provide information on potential allergies and other adverse health-related issues [31,36,39]. The latter issue is also important to consider for Quorn and other businesses involved in mycoprotein production [26].

Marketers of mycoproteins and other meat alternatives must thoroughly consider their targeting and profiling of potential consumers. When making comparisons between mycoprotein and traditional meat, marketers must consider their target consumers. Advertising nutritional and sensory similarity to meat may be appealing to consumers who enjoy eating traditional meat products, especially those who wish to reduce the meat in their diet for health reasons. However, such information may not be as suitable for people following a vegan or vegetarian diet. For those consumers, marketers should consider emphasizing the safety and sustainability aspects of mycoprotein and make comparisons with other plant-based products.

The data of the present study were procured using social media and email; however, the novelty of the topic and the comparison among consumers with different meat-eating behaviors add value to the recent body of literature. The non-probability-based nature of the sampling approach should be acknowledged. A social media sample was chosen to overcome budget constraints. In addition, the debate of whether to consume fungal- and plant-based meat alternatives can be controversial and sensitive, so a sampling approach via social media platforms was considered suitable for the present study overall, despite its limitations as a potential source of sampling bias. Nevertheless, social media platforms allow researchers to access their personal contacts who are members of interest groups that connect other users throughout the internet [52]. Such groups are classified as online communities connecting members with shared interests, attitudes, and, in the case of this study’s context, consumption habits. A multi-referral sampling approach can mitigate the risk of obtaining one-dimensional information from survey participants [52]. However, the sampling approach led to a sample that is relatively young, and the voices of elderly consumers may have been under-represented. Recruitment through a dietary organization or opt-panel providers in the future would allow for representative sampling and more specific consideration of socio-cultural background factors such as ethnicity or religion, as they are likely to impact consumption and access to the product in the market.

Future research may be dedicated to cross-country comparisons, as well as investigations related to mycoproteins and sustainability. Mycoproteins are praised for their contribution to the environmental component of sustainability (Quorn is certified for its contribution to a low carbon footprint) [41]; however, the other aspects of sustainability are widely disregarded. A best–worst approach will allow uncovering consumer preferences for varying sustainability product attributes. Further studies may be adapted from Lombardi et al., 2017 [53] and be dedicated to climate-neutral meals. Choice experiments to explore consumer preferences and willingness to accept would be suitable, as climate-friendly meals may require renunciation which would be accommodated in a willingness-to-accept scenario.

## Figures and Tables

**Table 1:** Demographic Information (*n* = 4888).

		Demographics			Meat-Eating Behavior		
Country	*n*	Male %	Female %	Age (Mean)	Omnivore	Flexitarian	Vegetarian
UK	758	71.1%	28.6%	31.1	67.9%	17.8%	14.2%
Pakistan	649	65.6%	34.1%	23.6	77.2%	15.3%	7.6%
China	556	37.9%	60.8%	31.2	78.6%	17.1%	4.3%
USA	521	75.6%	24.0%	44.0	66.8%	16.5%	16.7%
France	491	81.7%	18.1%	29.0	60.3%	31.6%	8.1%
New Zealand	259	54.1%	44.8%	38.6	75.7%	13.9%	10.4%
Netherlands	230	62.6%	37.4%	29.4	42.6%	40.9%	16.5%
Mexico	227	65.6%	33.9%	39.4	68.3%	29.5%	2.2%
Brazil	212	57.5%	42.5%	42.7	73.1%	21.2%	5.7%
Indonesia	210	55.2%	43.3%	35.6	89.0%	9.5%	1.4%
Spain	199	49.2%	48.7%	35.1	63.8%	32.7%	3.5%
Dominican Republic	176	65.3%	33.5%	26.2	67.6%	27.8%	4.5%
Total (percentage)		63.6%	35.8%		69.8%	21.1%	9.1%
Total (count/average)	4488	2855	1606	33.2	3134	946	408

**Table 2:** Descriptive Statistics for Single-Item Measures.

Scale	Mean	Min	Max	StDev	Means for Subgroups				
Mycoprotein Characteristics (1 = Strongly Disagree to 5 = Strongly Agree)					Male	Female	Omni	Flexi	Vege
Mycoprotein is healthy	3.85	1	5	0.84	3.75	3.83	3.71	3.85	4.21
Mycoprotein is safe to eat	3.72	1	5	0.90	3.63	3.74	3.59	3.72	4.19
Mycoprotein is nutritious	3.83	1	5	0.85	3.75	3.81	3.69	3.83	4.29
Mycoprotein is more sustainable	3.60	1	5	0.94	3.41	3.43	3.26	3.60	4.22
Mycoprotein is tasty	2.88	1	5	0.96	2.72	2.68	2.55	2.88	3.56
Mycoprotein is affordable	3.11	1	5	0.90	3.10	3.08	3.03	3.11	3.49
**Willingness to Consume (1 = No, 2 = Possible, 3 = Yes)**									
Willingness to Try Mycoprotein	2.01	1	3	0.82	2.40	2.41	2.33	2.52	2.66
Willingness to Buy Mycoprotein	1.73	1	3	0.74	2.17	2.20	2.08	2.33	2.63
Willingness to Pay More for Mycoprotein	1.39	1	3	0.60	1.62	1.64	1.51	1.76	2.19

**Table 3:** Scale Loadings, Reliabilities, and Convergent Validity.

Scales and Items	Overall		Means for Subgroups					Factor Loadings	Cronbach’s Alpha	Composite Reliability	AVE
	Mean	St Dev	Male	Female	Omni	Flexi	Vege				
**Nutritional Importance of Meat (1 = Strongly Disagree to 5 = Strongly Agree)**									0.858	0.934	0.875
Eating meat is necessary for obtaining beneficial nutrients	3.51	1.27	3.41	3.69	3.85	3.11	1.79	0.929			
Meat is an important part of a healthy and balanced diet	3.66	1.16	3.55	3.87	4.00	3.32	1.82	0.942			
**Sensory Importance of Meat** **(1 = Strongly Disagree to 5 = Strongly Agree)**									0.935	0.959	0.885
The taste of meat is important to me	4.02	1.11	3.91	4.23	4.35	3.80	2.04	0.949			
The texture of meat is important to me	3.95	1.12	3.86	4.11	4.25	3.75	2.04	0.952			
The smell of meat is important to me	3.85	1.12	3.75	4.04	4.16	3.65	1.95	0.922			

**Table 4:** Fornell–Larcker Criterion, and Heterotrait–Monotrait Ratio.

Fornell–Larcker Criterion	Nutritional Importance of Meat	Sensory Importance of Meat
Nutritional Importance of Meat	0.936	
Sensory Importance of Meat	0.627	0.941
**Heterotrait–Monotrait Ratio**		
Sensory Importance of Meat	0.696	

**Table 5:** Goodness of Fit, Explanatory Power, and Predictive Relevance Indices.

Model Indices	Overall	Male	Female	Omni	Flexi	Vege
Goodness of Fit	0.522	0.542	0.490	0.471	0.489	0.621
NFI	0.938	0.941	0.927	0.919	0.922	0.943
SRMR	0.021	0.019	0.030	0.030	0.026	0.016
Explanatory Power (Average R^2^)	0.272	0.294	0.240	0.222	0.239	0.385
Predictive Relevance (Average Q^2^)	0.268	0.288	0.230	0.216	0.219	0.366

**Table 6:** Results of Hypothesis Testing.

Sample/Subsample	Complete Sample			Male Subgroup			Female Subsample			Omnivore Subsample			Flexitarian Subsample			Vegetarian Subsample		
Hypothesized Path Relationship	Coefficient	t-Stat	*p* Value	Coefficient	t-Stat	*p* Value	Coefficient	t-Stat	*p* Value	Coefficient	t-Stat	*p* Value	Coefficient	t-Stat	*p* Value	Coefficient	t-Stat	*p* Value
H1a: Healthy -> WtT	**0.161**	5.830	0.000	**0.204**	5.969	0.000	0.073	1.594	0.111	**0.185**	5.591	0.000	0.099	1.665	0.096	0.013	0.154	0.877
H1b: Healthy -> WtB	**0.194**	8.001	0.000	**0.216**	7.355	0.000	**0.152**	3.537	0.000	**0.227**	7.623	0.000	**0.149**	2.835	0.005	−0.001	0.016	0.987
H1c: Healthy -> WtPM	**0.079**	3.422	0.001	**0.064**	2.285	0.022	**0.108**	2.715	0.007	**0.101**	3.757	0.000	0.051	0.964	0.335	0.041	0.387	0.699
H2a: Safe to Eat -> WtT	**0.115**	4.772	0.000	**0.100**	3.334	0.001	**0.135**	3.295	0.001	**0.107**	3.760	0.000	**0.103**	1.990	0.047	**0.215**	2.814	0.005
H2b: Safe to Eat -> WtB	**0.129**	5.615	0.000	**0.112**	4.054	0.000	**0.155**	3.831	0.000	**0.103**	3.736	0.000	**0.201**	4.218	0.000	**0.205**	2.703	0.007
H2c: Safe to Eat -> WtPM	**0.068**	3.283	0.001	**0.076**	2.908	0.004	0.045	1.289	0.198	**0.050**	2.016	0.044	0.078	1.597	0.110	**0.182**	2.106	0.035
H3a: Nutritious -> WtT	**0.138**	5.253	0.000	**0.109**	3.299	0.001	**0.196**	4.611	0.000	**0.145**	4.769	0.000	**0.153**	2.703	0.007	0.082	0.874	0.382
H3b: Nutritious -> WtB	**0.125**	5.433	0.000	**0.117**	4.104	0.000	**0.137**	3.416	0.001	**0.137**	5.090	0.000	0.090	1.727	0.084	0.156	1.747	0.081
H3c: Nutritious -> WtPM	**0.055**	2.610	0.009	**0.058**	2.241	0.025	0.058	1.677	0.094	**0.052**	2.209	0.027	0.076	1.451	0.147	0.008	0.069	0.945
H4a: Taste -> WtT	**−0.042**	2.338	0.019	−0.030	1.379	0.168	**−0.064**	2.069	0.039	−0.031	1.496	0.135	−0.044	1.156	0.248	−0.069	1.434	0.152
H4b: Taste -> WtB	**0.057**	3.347	0.001	**0.055**	2.693	0.007	**0.060**	2.078	0.038	**0.067**	3.351	0.001	0.038	1.027	0.304	−0.003	0.061	0.951
H4c: Taste -> WtPM	**0.255**	15.689	0.000	**0.254**	12.322	0.000	**0.262**	9.868	0.000	**0.283**	14.710	0.000	**0.227**	6.253	0.000	0.085	1.594	0.111
H5a: Affordability -> WtT	**−0.070**	4.469	0.000	**−0.061**	3.175	0.002	**−0.082**	3.048	0.002	**−0.079**	4.052	0.000	−0.040	1.176	0.240	**−0.076**	2.059	0.040
H5b: Affordability -> WtB	−0.030	1.949	0.051	−0.020	1.117	0.264	−0.043	1.586	0.113	−0.030	1.573	0.116	−0.016	0.481	0.630	−0.063	1.692	0.091
H5c: Affordability -> WtPM	0.008	0.481	0.631	0.006	0.289	0.772	0.003	0.118	0.906	0.010	0.538	0.591	−0.020	0.536	0.592	0.081	1.414	0.157
H6a: Sustainable -> WtT	**0.106**	5.857	0.000	**0.121**	5.149	0.000	**0.086**	2.950	0.003	**0.079**	3.856	0.000	**0.182**	4.685	0.000	**0.190**	2.862	0.004
H6b: Sustainable -> WtB	**0.074**	4.194	0.000	**0.094**	4.354	0.000	0.040	1.396	0.163	**0.051**	2.496	0.013	**0.111**	3.018	0.003	**0.196**	3.342	0.001
H6c: Sustainable -> WtPM	**0.058**	3.408	0.001	**0.061**	2.822	0.005	0.047	1.582	0.114	0.031	1.546	0.122	**0.099**	2.606	0.009	0.117	1.645	0.100
H7a: Nutritional Importance of Meat -> WtT	**−0.186**	11.212	0.000	**−0.181**	8.307	0.000	**−0.185**	7.279	0.000	**−0.147**	8.565	0.000	**−0.155**	4.905	0.000	**−0.379**	5.494	0.000
H7b: Nutritional Importance of Meat -> WtB	**−0.171**	10.720	0.000	**−0.165**	8.007	0.000	**−0.182**	7.463	0.000	**−0.122**	7.389	0.000	**−0.159**	5.166	0.000	**−0.322**	5.265	0.000
H7c: Nutritional Importance of Meat -> WtPM	**−0.101**	5.783	0.000	**−0.118**	5.272	0.000	**−0.080**	2.915	0.004	−0.032	1.686	0.092	**−0.127**	3.705	0.000	**−0.161**	2.718	0.007
H8a: Sensory Importance of Meat -> WtT	0.015	0.954	0.340	0.006	0.298	0.766	0.038	1.513	0.130	−0.004	0.256	0.798	0.001	0.041	0.967	−0.018	0.340	0.734
H8b: Sensory Importance of Meat -> WtB	**−0.043**	2.745	0.006	**−0.061**	3.062	0.002	−0.009	0.389	0.697	**−0.050**	3.085	0.002	−0.020	0.655	0.512	−0.018	0.348	0.728
H8c: Sensory Importance of Meat -> WtPM	**−0.123**	7.086	0.000	**−0.109**	4.845	0.000	**−0.160**	6.118	0.000	**−0.100**	5.527	0.000	−0.027	0.823	0.410	−0.049	0.897	0.370

Bold: significant at *p* ≥ 0.05; orange cell: significant in complete sample, but n.s. in subsample.

## Data Availability

The data presented in this study are available on request from the corresponding author.

## Supplementary Materials

The following supporting information can be downloaded at: https://www.mdpi.com/article/10.3390/nu14163292/s1, Supplementary S1: Survey Questions
