# Supplementary material for: Understanding Key Factors Influencing Consumers’ Willingness to Try, Buy, and Pay a Price Premium for Mycoproteins
Source: Nutrients. 2022 Aug 11;14(16):3292. doi: 10.3390/nu14163292 (PMC9416216; doi:10.3390/nu14163292)
Supplement: Supplementary file 1 [file nutrients-14-03292-s001.zip › nutrients-1836539-supplementary.pdf]

Survey Questions

Gender
Male =1 Female =2

Age
Your age (in years)

Meat Eating Behaviour
Do you actively avoid meat or other animal products from your diet?
1= I avoid meat some days (Flexitarian)
2= Yes (Vegetarian)
3= No (Omnivore)

Fungal-based Protein Characteristics (1=Strongly Disagree to 5=Strongly Agree)
Fungal-based protein is healthy
Fungal-based protein is safe to eat
Fungal-based protein is nutritious
Fungal-based protein is more sustainable
Fungal-based protein is tastier
Fungal-based protein is cheaper

Willingness To Consume (1=No, 2=Possible, 3=Yes)
I am willing to try fungal-based protein
I am willing to purchase fungal-based protein
I am willing to pay more for fungal-based protein

Nutritional Importance of Meat (1=Strongly Disagree to 5=Strongly Agree)
Eating meat is necessary for obtaining beneficial nutrients
The nutritional benefits of meat can easily be matched by alternative protein sources
Meat is an important part of a healthy and balanced diet

Sensory Importance of Meat (1=Strongly Disagree to 5=Strongly Agree)
The taste of meat is important to me
The texture of meat is important to me
The smell of meat is important to me
